# Vision-Based Obstacle Avoidance Strategies for MAVs Using Optical Flows in 3-D Textured Environments

**DOI:** 10.3390/s19112523

**Published:** 2019-06-02

**Authors:** Gangik Cho, Jongyun Kim, Hyondong Oh

**Affiliations:** School of Mechanical, Aerospace and Nuclear Engineering, Ulsan National Institute of Science and Technology, 50, UNIST-gil, Banyeon-ri, Eonyang-eup, Ulju-gun, Ulsan 44919, Korea; chogi89@unist.ac.kr (G.C.); jykim17@unist.ac.kr (J.K.)

**Keywords:** vision-based obstacle avoidance, optical flow, Horn-Schunck method, focus of expansion, micro aerial vehicle

## Abstract

Due to payload restrictions for micro aerial vehicles (MAVs), vision-based approaches have been widely studied with their light weight characteristics and cost effectiveness. In particular, optical flow-based obstacle avoidance has proven to be one of the most efficient methods in terms of obstacle avoidance capabilities and computational load; however, existing approaches do not consider 3-D complex environments. In addition, most approaches are unable to deal with situations where there are wall-like frontal obstacles. Although some algorithms consider wall-like frontal obstacles, they cause a jitter or unnecessary motion. To address these limitations, this paper proposes a vision-based obstacle avoidance algorithm for MAVs using the optical flow in 3-D textured environments. The image obtained from a monocular camera is first split into two horizontal and vertical half planes. The desired heading direction and climb rate are then determined by comparing the sum of optical flows between half planes horizontally and vertically, respectively, for obstacle avoidance in 3-D environments. Besides, the proposed approach is capable of avoiding wall-like frontal obstacles by considering the divergence of the optical flow at the focus of expansion and navigating to the goal position using a sigmoid weighting function. The performance of the proposed algorithm was validated through numerical simulations and indoor flight experiments in various situations.

## 1. Introduction

To navigate through unknown environments, micro aerial vehicles (MAVs) need to detect and avoid obstacles. There are many types of sensors to detect obstacles such as RADAR (RAdio Detection And Ranging), LiDAR (LIght Detection And Ranging), and ultrasonic sensor. RADAR and LiDAR have a good performance in terms of the operation range and accuracy to detect obstacles around the MAV. However, they have disadvantages in terms of operating time due to heavy weight and energy consumption. Although lightweight and low power consumption products have been developed recently [1], they are still expensive. Ultrasonic sensors are small and light but typically have poor range accuracy. Vision sensors, on the other hand, have various advantages such as lightweight, large detection area, fast response time, low cost, and rich information about the environment around the MAV.

There are various obstacle avoidance methods using the vision system. Tomoyuki et al. [2] and Abdulla et al. [3] conducted obstacle avoidance experiment with a monocular camera using the feature match algorithms such as SURF (Speeded-Up Robust Features) and SIFT (Scale-Invariant Feature Transform). The algorithms extract the descriptors from the obstacles and detect them using the expansion of descriptors. However, they performed the experiments with the off-board computer (i.e., desktop PC) since the feature matching algorithm demands a heavy computational power. On the other hand, optical flow-based obstacle avoidance methods can be readily applicable to the lightweight on-board computer of the MAV as they require much less computational power compared to the feature-based algorithms [4,5,6,7,8,9,10,11,12,13,14]. Souhila et al. implemented an obstacle avoidance strategy for the ground vehicle by changing a heading angle according to the amount of the optical flow difference between the left and right half planes of the image, called the balance strategy [4]. Yoo et al. applied the balance strategy to the multi-rotor UAV and carried out simulations in a virtual 3-D space [12]. Eresen et al. made the UAV detect obstacles and junctions using the optical flow and turn at a junction in the Google Earth environment [11]. The limitation of the balance strategy is that the MAV cannot recognize obstacles where the optical flow difference between the two half planes of the image is small. To overcome this limitation, Agrawal et al. designed a steering command inversely proportional to the optical flow difference so that the UAV turns around in front of a wall-like obstacle [7]. However, this approach could cause jitter in the case of low optical flow difference. Muratet et al. developed the algorithm to detect a frontal wall-like obstacle by using the amount of the expansion of the optical flow [5,13]. If the frontal obstacle is detected, then the UAV decreases its speed or makes a U-turn. Prashanth et al. developed a logic to avoid obstacles by changing the pitch angle based on the optical flow difference between top and bottom halves of the image [14]; however, they did not carry out simulations or flight experiments.

In this paper, we propose a vision-based obstacle avoidance algorithm based on the horizontal balance strategy using the optical flow. Along with horizontal avoidance, it is designed to change the height to avoid the obstacle vertically by balancing the optical flow generated in the upper and lower half plane of the image. Besides, if a wall-like obstacle is in front of the MAV directly, the MAV turns the heading angle according to the expansion of the optical flow. Previously, the MAV was made to turn inversely proportional to the optical flow difference [7] or take a U-turn in case of a large amount of the expansion of the optical flow [5,13]. Meanwhile, the proposed algorithm uses a proportional-derivative (PD) controller with the yaw rate compensator according to the amount of the expansion of the optical flow so that the MAV can avoid the frontal obstacle without the jitter or unnecessary U-turns. The proposed algorithm also includes the guidance to the goal position or waypoint if there are no obstacles around. The proposed algorithm combines above obstacle avoidance strategies and waypoint guidance with appropriate weights according to the environment condition so that the MAV avoids obstacles in 3-D environments while moving towards the goal position. The performance of the proposed algorithm was verified by the numerical simulations using the RotorS simulator running in the Robot Operating System (ROS) and the Gazebo environment and indoor flight experiments using the quadrotor MAV.

The contribution of this paper is threefold: (i) the MAV avoids the obstacle by changing the height as well as heading angle in 3-D environments; (ii) the MAV can avoid the frontal obstacle which generates a similar optical flow in left and right half planes such as a wall by using the expansion of the optical flow; and (iii) the MAV combines the different guidance strategies with adequate weights considering the environment around the MAV.

This rest of this paper is organized as follows. In Section 2, the computation of the optical flow is presented. In Section 3, obstacle avoidance strategies according to the position of the obstacle and the waypoint guidance strategy are proposed. The proposed obstacle avoidance algorithm was verified with numerical simulations and indoor flight experiments, which are presented in Section 4 and Section 5, respectively.

## 2. Optical Flow

The optical flow refers to the movement of each pixel expressed in the image plane when an object moving in the three-dimensional space is projected onto the two-dimensional image plane. There are various methods to estimate the optical flow such as [15,16,17]. Recently, learning-based optical flow estimation methods are also proposed [3,18,19]. In this work, the optical flow computation method proposed by Horn and Schunck [16] is adopted. The concept for computing optical flows is briefly introduced in the following for the sake of the completeness of the paper and readers’ convenience.

**Figure 1:**
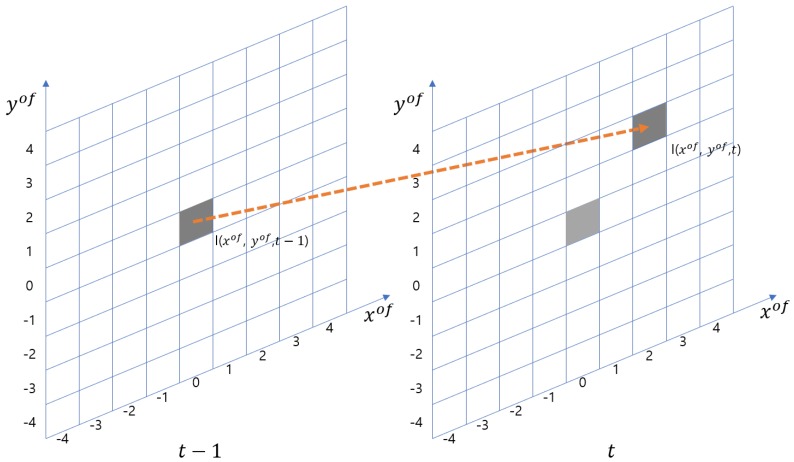
The intensity of a point is the same as time goes from t−1 to *t*. For instance, in this figure, I(0,0,t−1)=I(2,2,t).

In the Horn–Schunck method, two assumptions are used to estimate the optical flow. First, as in Figure 1, the intensity of a particular point is constant over time.

(1) $$\frac{dI(x^{of},y^{of},t)}{dt}=0$$

where *I* is the intensity of the pixel at the point $\left(x^{of},y^{of}\right)$. By using the chain rule, Equation (1) can be expressed as:

(2) $$I_{x}\frac{dx^{of}}{dt}+I_{y}\frac{dy^{of}}{dt}+I_{t}=0$$

where $I_{x}$, $I_{y}$ and $I_{t}$ are partial derivatives of the intensity at each pixel with respect to $x^{of}$, $y^{of}$ axes and time *t*, respectively. By defining the optical flow vector (velocity vector of the pixels) as $V:=\left(u,v\right)=\left({\dot{x}}^{of},{\dot{y}}^{of}\right)$, Equation (2) is expressed in a different form:

(3) $$I_{t}=-\left(I_{x}{\dot{x}}^{of}+I_{y}{\dot{y}}^{of}\right)=-\left(I_{x}u+I_{y}v\right)=-\nabla I\cdot V$$

where $\nabla I={\left(I_{x},I_{y}\right)}^{T}$, *u* and *v* are $x^{of}$ and $y^{of}$ directions of the optical flow, respectively. The cost function to minimize the change of the intensity over the time is defined by:

(4) $$J_{b}=I_{t}+\nabla I\cdot V.$$

The second assumption is a smoothness constraint, which represents that the neighboring points on an object have a similar optical flow. Having a similar optical flow at the neighboring points implies that the magnitude of the gradient of all optical flows should be minimized. It can be expressed by:

(5) $$J_{s}^{2}={\left(\frac{\partial u}{\partial x^{of}}\right)}^{2}+{\left(\frac{\partial u}{\partial y^{of}}\right)}^{2}+{\left(\frac{\partial v}{\partial x^{of}}\right)}^{2}+{\left(\frac{\partial v}{\partial y^{of}}\right)}^{2}.$$

The total cost function to be minimized is designed using Equations (4) and (5) by

(6) $$J=\underset{r}{\;\int \int \;}\left(\alpha^{2}J_{s}^{2}+J_{b}^{2}\right)dxdy$$

where α is a weighting factor for the smoothness constraint. Then, *u* and *v* minimizing Equation (6), which are the optical flow at each pixel, can be obtained:

(7) $$\begin{array}{ccc} u & = & \bar{u}-I_{x}\frac{I_{x}\bar{u}+I_{y}\bar{v}+I_{t}}{\alpha^{2}+I_{x}^{2}+I_{y}^{2}}, \end{array}$$

(8) $$\begin{array}{ccc} v & = & \bar{v}-I_{y}\frac{I_{x}\bar{u}+I_{y}\bar{v}+I_{t}}{\alpha^{2}+I_{x}^{2}+I_{y}^{2}}. \end{array}$$

From Equations (7) and (8), an iterative solution can be obtained as:

(9) $$\begin{array}{c} u^{k+1}={\bar{u}}^{k}-I_{x}\frac{I_{x}{\bar{u}}^{k}+I_{y}{\bar{v}}^{k}+I_{t}}{\alpha^{2}+I_{x}^{2}+I_{y}^{2}}, \end{array}$$

(10) $$\begin{array}{c} v^{k+1}={\bar{v}}^{k}-I_{y}\frac{I_{x}{\bar{u}}^{k}+I_{y}{\bar{v}}^{k}+I_{t}}{\alpha^{2}+I_{x}^{2}+I_{y}^{2}}. \end{array}$$

where $u^{k+1}$ and $v^{k+1}$ are the optical flow of the $x^{of}$ and $y^{of}$ axes at the k+1th time frame, respectively.

## 3. Obstacle Avoidance Strategy

This section introduces the obstacle avoidance strategies using the optical flow estimated from a forward looking monocular camera. The horizontal balance strategy vertical balance strategy to avoid the obstacle vertically, and the frontal obstacle avoidance strategy which exploits the expansion of the optical flow are introduced. In addition to obstacle avoidance strategies, the waypoint guidance strategy is described to reach to the goal point for the MAV. Lastly, the above strategies are integrated with the weighted sum of guidance commands from different strategies according to obstacle environment conditions.

### 3.1. Horizontal Balance Strategy

This subsection deals with the horizontal balance strategy. We modified conventional balance strategies [5,20,21] using the concept of the proportional-derivative (PD) controller. First, we show how the magnitude of the optical flow changes as the MAV approaches to an obstacle, and then the methodology of the horizontal balance strategy is introduced.

**Figure 2:**
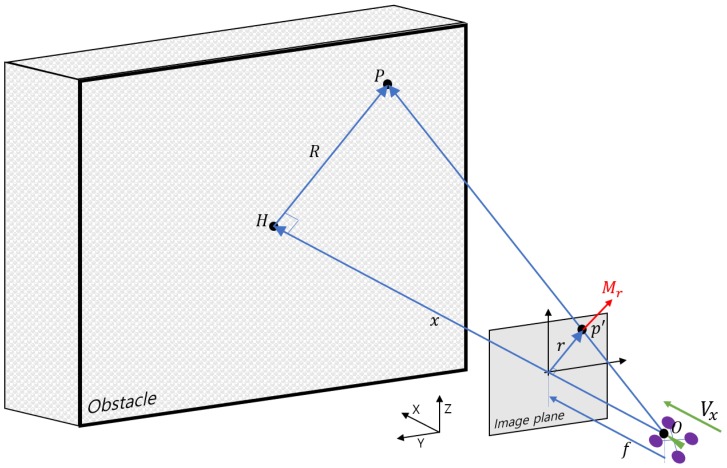
Geometry to compute the optical flow for an arbitrary point *P* in the obstacle.

As shown in Figure 2, suppose there is the point *P* on the obstacle of interest corresponding to the point $p^{\prime }$ on the image plane. The vector R is defined as the vector from the nearest point *H* on the principal axis of the camera to the point *P*. The vector r is the vector corresponding to R on the image plane. The MAV approaches the obstacle with the forward speed $V_{x}$ at the point *O*. Note that the focal length *f* and $\bar{OH}=x$ are given. Considering the geometrical relationship, the following relation holds:

(11) $$\frac{r(t)}{f}=\frac{R}{x(t)}.$$

It is worth noting that r and *x* are functions of time *t* and *f* and R does not change as the MAV moves. Differentiating Equation (11) with respect to time gives:

(12) $$\frac{\dot{r}}{f}=-R\frac{\dot{x}}{x^{2}}.$$

By rewriting Equation (12), the optical flow $\dot{r}=M_{r}$ for a particular point of the obstacle is obtained as:

(13) $$M_{r}=\frac{fRV_{x}}{x^{2}}$$

where the speed of the MAV $V_{x}=-\dot{x}$. In Equation (13), the denominator is a function of time, whereas the numerator is constant with the assumption that the MAV keeps a constant speed. Note that the magnitude of the optical flow $M_{r}$ increases as the MAV approaches the obstacle.

**Figure 3:**
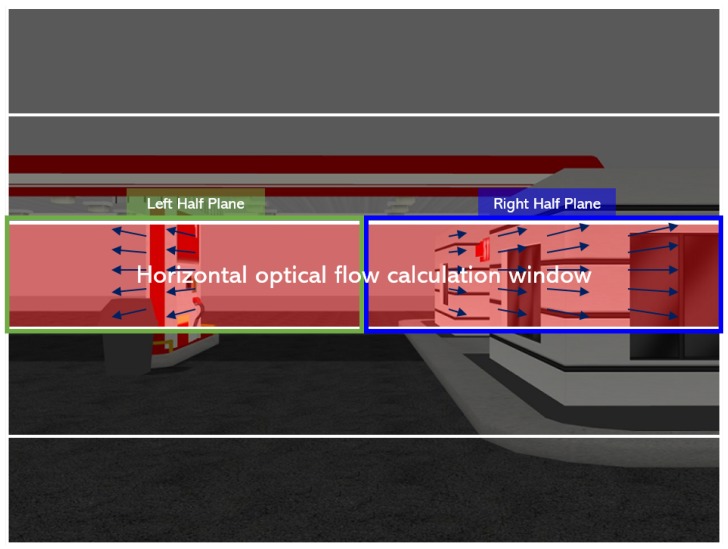
Horizontal optical flow calculation window.

Let the image be equivalently split into two horizontal (left and right) and vertical (upper and lower) half planes. Considering the fact that the larger the magnitude of the optical flow is, the closer the obstacle is, the desired heading direction of the MAV can be determined by finding the half plane which has the smaller sum of optical flows. More specifically, the optical flow is calculated within the horizontal calculation window shown as the red area in Figure 3 to ignore the unnecessary part of the environment where collision does not need to be considered. The horizontal optical flow calculation window is the center region among five evenly-divided regions of the image plane horizontally. The size and position of the horizontal optical flow calculation window were empirically determined. The heading rate command (${\dot{\psi }}_{d}^{rl}$) is generated by the PD controller with the error ($e_{rl}$) defined as the difference of the sum of the optical flows at left ($M_{left}=\sum_{left}\sqrt{u_{i}^{2}+v_{i}^{2}}$) and right ($M_{right}=\sum_{right}\sqrt{u_{i}^{2}+v_{i}^{2}}$) half planes, as shown in Figure 3:

(14) $${\dot{\psi }}_{d}^{rl}=k_{P,rl}e_{rl}+k_{D,rl}{\dot{e}}_{rl}$$

where $k_{P,rl}$ and $k_{D,rl}$ are positive gains for the horizontal balance strategy. Gain values were set to $k_{P,rl}=0.01$ and $k_{D,rl}=0.01$ in the numerical simulations presented in Section 4, and $k_{P,rl}=0.007$ and $k_{D,rl}=0.001$ in the indoor flight experiments presented in Section 5.

### 3.2. Vertical Balance Strategy

**Figure 4:**
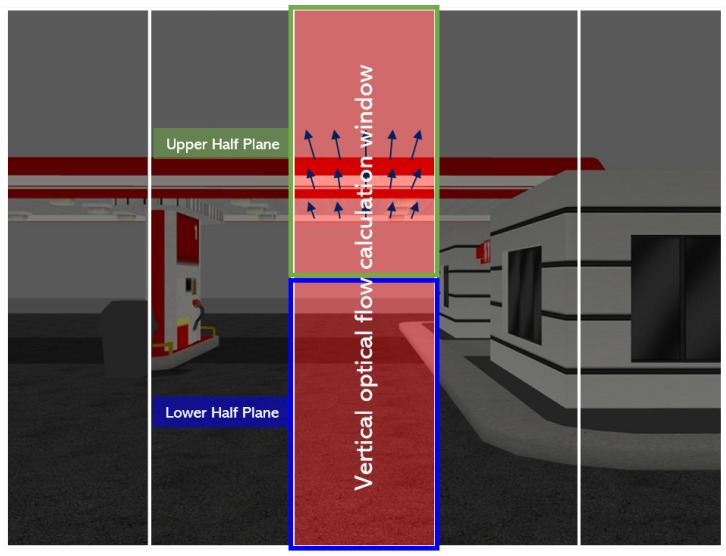
Vertical optical flow calculation window.

To avoid the obstacle in the three-dimensional environment, the MAV has to change the altitude as well as heading angle. In this subsection, the obstacle avoidance strategy by changing the altitude of the MAV is proposed. The optical flow for the vertical balance strategy is calculated within the vertical calculation window shown as the red area in Figure 4 where the unnecessary part to avoid the obstacle is ignored. The vertical optical flow calculation window is obtained similarly to the horizontal calculation window by dividing the image plane vertically. The size and position of the vertical optical flow calculation window were empirically determined.

The desired climb rate command (${\dot{h}}_{d}$) can be generated by the PD controller with the error ($e_{ud}\;=\;M_{down}-M_{up}$), which is the difference of the sum of optical flows at lower ($M_{down}$) and upper ($M_{up}$) half planes shown in Figure 4. However, when the MAV moves with a high climb rate, the additional optical flow could be generated due to the effect of vertical movements, and, as a result, the error $e_{ud}$ could diverge unexpectedly. To avoid the unwanted effect from $\dot{h}$, the error $e_{ud}$ for the PD controller is modified to:

(15) $$e_{m,ud}=\frac{e_{ud}}{1+k_{P,m}\left|\dot{h}\right|}$$

where $k_{P,m}=$ 1000 is the weighting for the magnitude of the climb rate. In Equation (15), if $\dot{h}=0$, then, $e_{m,ud}$ is equal to $e_{ud}$ and the larger the climb rate is, the closer the $e_{m,ud}$ is to zero so that the MAV does not change the altitude much. The PD controller for vertical balance strategy can then be defined as:

(16) $${\dot{h}}_{d}=k_{P,ud}e_{m,ud}+k_{D,ud}{\dot{e}}_{m,ud}$$

where $k_{P,ud}$ and $k_{D,ud}$ are positive gains of the vertical balance strategy. Gain values were set to $k_{P,ud}=0.01$ and $k_{D,ud}=0.01$ in the numerical simulations presented in Section 4, and $k_{P,ud}=0.03$ and $k_{D,ud}=0.01$ in the indoor flight experiments presented in Section 5.

### 3.3. Frontal Obstacle Avoidance Strategy

**Figure 5:**
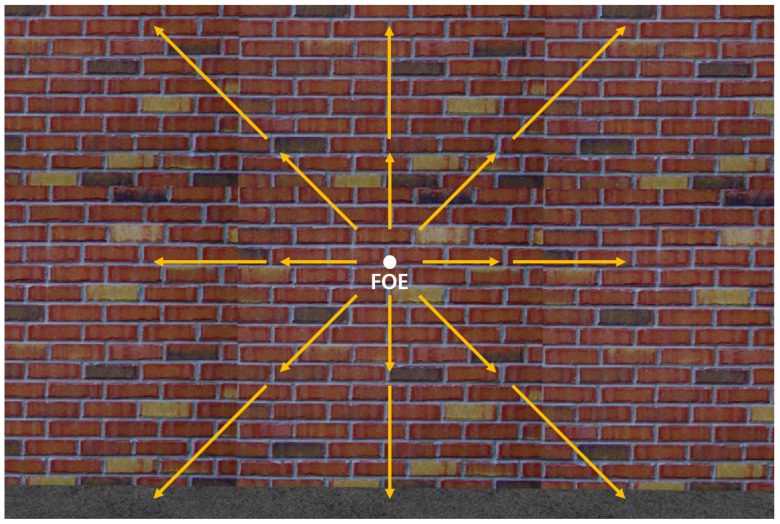
The optical flows diverge from the FOE when the MAV goes towards the obstacle directly.

Horizontal and vertical balance obstacle avoidance strategies might not work when the obstacle is directly in front of the MAV. In this situation, the optical flows on both the half planes are similar, so the MAV would go forward without changing the heading angle and collide with the obstacle. To address this problem, the concept of expansion of the optical flow at the focus of expansion (FOE) is introduced in [5]. When the MAV goes towards the wall-like obstacle directly, the diverging optical flow is generated, as shown in Figure 5, where the optical flows expand from the FOE close to the origin of the image.

Using the geometry given in Figure 2, by assuming that FOE and principal point of the image plane coincide and the optical flow expands from the FOE, the following relation can be obtained:

(17) $$\frac{∥M_{r}∥}{∥r∥}=\frac{V_{x}}{x}=\frac{1}{\tau }$$

where time-to-contact τ can be expressed by the ratio between $V_{x}$ and *x*. When $V_{x}$ is large or *x* is small, the time-to-contact becomes small. Using this relation, the time-to-contact τ can be computed. We define the inverse of τ as η, expansion of the optical flow (EOF). Since $M_{r}$ has the same direction with r, the EOF can be computed in a vector form as:

(18) $$\eta =\frac{1}{\tau }=\frac{M_{r}\cdot r}{{∥r∥}^{2}}.$$

Note that the high EOF implies that there is a high risk of the collision with the frontal obstacle. The magnitude of the desired heading rate to avoid the frontal obstacle is determined by the PD controller using the EOF as:

(19) $$\begin{array}{ccc} {\dot{\psi }}_{d}^{\eta } & = & sign\left(e_{\eta }\right)\left(k_{P,\eta }\eta_{sum}+k_{D,\eta }{\dot{\eta }}_{sum}\right), \end{array}$$

(20) $$\begin{array}{ccc} \eta_{sum} & = & \sum_{N}^{i=1}\eta_{i}, \end{array}$$

where $e_{\eta }$ ($=\eta_{sum}^{right}-\eta_{sum}^{left}$) is the difference between the sum of EOF in the left and right half planes. $\eta_{sum}$ is the sum of the EOF in the horizontal optical flow calculation window and *N* is the number of pixels in the horizontal optical flow calculation window. $k_{P,\eta }$ and $k_{D,\eta }$ are positive gains of the frontal obstacle avoidance controller. Gain values were set to $k_{P,\eta }=0.8$ and $k_{D,\eta }=0.01$ in numerical simulations presented in Section 4, and $k_{P,\eta }=1.2$ and $k_{D,\eta }=0.01$ in the indoor flight experiments presented in Section 5. The sign of $e_{\eta }$ determines the turning direction, which makes the MAV rotate to the opposite direction in which the obstacle is most likely to be close.

### 3.4. Waypoint Guidance

In addition to the obstacle avoidance, the MAV may need to reach the waypoint to accomplish the given mission. The heading rate and climb rate for waypoint guidance can be determined as:

(21) $$\begin{array}{ccc} {\dot{\psi }}_{d}^{wp} & = & k_{P,wp,\psi }e_{\psi }+k_{D,wp,\psi }{\dot{e}}_{\psi }, \end{array}$$

(22) $$\begin{array}{ccc} {\dot{h}}_{d}^{wp} & = & k_{P,wp,h}e_{h}+k_{D,wp,h}{\dot{e}}_{h}, \end{array}$$

where the error ($e_{\psi }$) for the heading rate controller is the angle between the line from the current position to the waypoint and the current heading angle of the MAV $\psi_{c}$:

(23) $$e_{\psi }=\psi_{wp}-\psi_{c}.$$

The error for the climb rate is the difference between the height of the waypoint and the MAV:

(24) $$e_{h}=h_{wp}-h_{c}.$$

### 3.5. Hybrid Obstacle Avoidance Strategy

The hybrid obstacle avoidance strategy is designed to avoid various obstacles in 3-D environment while moving towards the goal according to existence of obstacles determined by the EOF η. The obstacle environment can be classified into three cases: (i) there is no obstacle around the MAV; (ii) there are obstacles but not directly in front of the MAV; and (iii) there are obstacles directly in front of the MAV. According to the classified situation, the obstacle avoidance strategies and waypoint guidance are combined with appropriate weighting factors. The weighting factors are designed with the sigmoid function according to the sum of the EOF:

(25) $$\begin{array}{ccc} \sigma_{rl} & = & \frac{1}{1+e^{k_{eta}\left(\eta_{sum}-\eta_{eta,0}\right)}}, \end{array}$$

(26) $$\begin{array}{ccc} \sigma_{eta} & = & \frac{1}{1+e^{-k_{eta}\left(\eta_{sum}-\eta_{eta,0}\right)}}, \end{array}$$

(27) $$\begin{array}{ccc} \sigma_{wp} & = & \frac{1}{1+e^{k_{wp}\left(\eta_{sum}-\eta_{wp,0}\right)}}. \end{array}$$

where $\eta_{eta,0}$ and $\eta_{wp,0}$ represent threshold values and $k_{eta}$ and $k_{wp}$ are positive gains.

**Figure 6:**
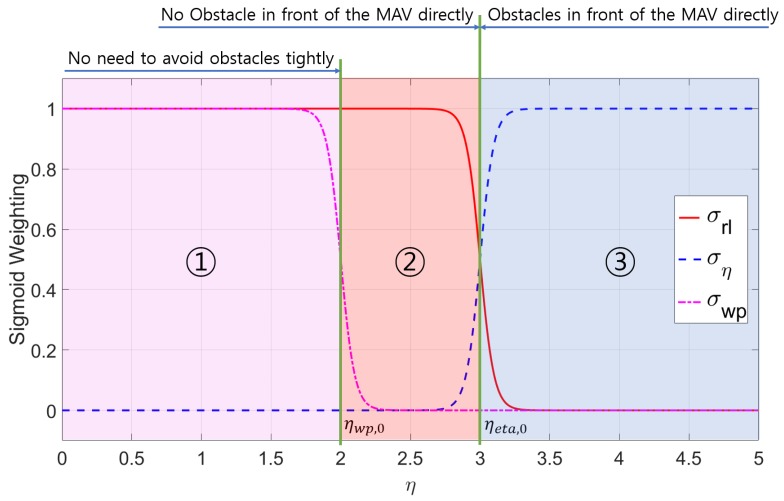
Sigmoid weighting with respect to the EOF.

Figure 6 shows the sigmoid weighting for determining the strategy with $k_{eta}=20$, $\eta_{eta,0}=3$, $k_{wp}=20$, and $\eta_{wp,0}=2$. Sigmoid weights largely contain three sections as the EOF changes. First, in the case that there is no obstacle around the MAV, the weighting for the balance strategy and waypoint guidance are activated and the MAV aims to the waypoint while avoiding the obstacle around the MAV. In the case that there is an obstacle around the MAV, the MAV first need to focus on avoiding the obstacle rather than moving to the waypoint. The MAV will avoid the obstacle not using waypoint guidance but just using the balance strategy. The final situation is that there is an obstacle in front of the MAV where the frontal obstacle avoidance strategy should be used. That is to say, either balance strategy or frontal obstacle avoidance strategy is always activated, and obstacle avoidance strategies are switched depending on the presence or absence of an obstacle directly in front of the MAV. Additionally, waypoint guidance is deactivated if there is a risk of collision and the MAV is required to focus on obstacle avoidance (i.e., $\eta >\eta_{wp,0}$). The gains for the sigmoid function were empirically determined.

The hybrid obstacle avoidance algorithm including waypoint guidance can then be designed as:

(28) $$\begin{array}{ccc} {\dot{\psi }}_{d} & = & \sigma_{rl}{\dot{\psi }}_{d}^{rl}+\sigma_{eta}{\dot{\psi }}_{d}^{eta}+\sigma_{wp}{\dot{\psi }}_{d}^{wp}, \end{array}$$

(29) $$\begin{array}{ccc} {\dot{h}}_{d} & = & {\dot{h}}_{d}^{ud}+{\dot{h}}_{d}^{wp}. \end{array}$$

Note that, unlike desired heading rate, the desired climb rate can be designed by just adding the vertical balance strategy and waypoint guidance without sigmoid weights. The sigmoid weights should not be multiplied to the vertical balance strategy considering the fact that the sigmoid weights are to change the heading angle of the MAV if there is a wall-like obstacle in front of the MAV.

## 4. Numerical Simulations

### 4.1. Simulation Environment Setup

The RotorS simulator [22] was exploited to verify the performance of the obstacle avoidance strategy. The RotorS simulator provides various multi-rotor helicopter models such as the AscTec Hummingbird, Pelican, and Firefly in the Gazebo environment. There are simulated sensors coming with the simulator such as an IMU, a generic odometry sensor, and the VI (Visual-Inertial) sensor, which can be mounted on the multi-rotor helicopters [23]. The RotorS simulator runs on the Robot Operating System (ROS). ROS is a flexible framework for operating various robot software. It is a collection of tools, libraries, and conventions that aim to simplify the task of creating complex and robust robot behavior across a wide variety of robotic platforms [24]. The RotorS simulator receives the desired command using ROS topics.

**Figure 7:**
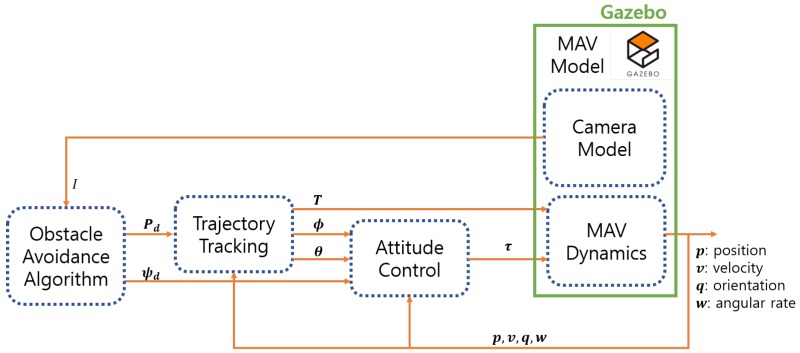
Obstacle avoidance simulator structure.

Figure 7 describes the obstacle avoidance simulator structure including RotorS simulator. It uses the desired position $P_{d}$ and the desired heading angle $\psi_{d}$ as guidance and control inputs. Using the desired position, trajectory tracking module generates the throttle command *T*, the roll angle command ϕ, and the pitch angle command θ. The attitude controller then generates a moment matrix τ in the body frame. The MAV dynamics makes the MAV move using the throttle and the moment, and provides states that consist of position *p*, velocity *v*, orientation *q* in the quaternion form, and angular velocity *w*. Using the obstacle avoidance algorithm, the desired position $P_{d}$ and heading angle $\psi_{d}$ are determined as:

(30) $$\begin{array}{ccc} P_{d} & = & \left[\begin{array}{c} P_{d,x}\\ P_{d,y}\\ P_{d,z} \end{array}\right]=\left[\begin{array}{c} P_{c,x}+r_{c}\cos\psi_{d}dt\\ P_{c,y}+r_{c}\sin\psi_{d}dt\\ P_{c,z}+{\dot{h}}_{d}dt \end{array}\right], \end{array}$$

(31) $$\begin{array}{ccc} \psi_{d} & = & \psi_{c}+{\dot{\psi }}_{d}dt. \end{array}$$

where $P_{d,x}$, $P_{d,y}$, and $P_{d,z}$ are the 3-D desired position; $P_{c,x}$, $P_{c,y}$, and $P_{c,z}$ are the current 3-D position; $\psi_{c}$ is the current heading angle; $r_{c}$ is a constant forward distance which can be determined by the forward speed of the MAV; and dt is the sampling frequency.

### 4.2. Simulations Result

**Figure 8:**
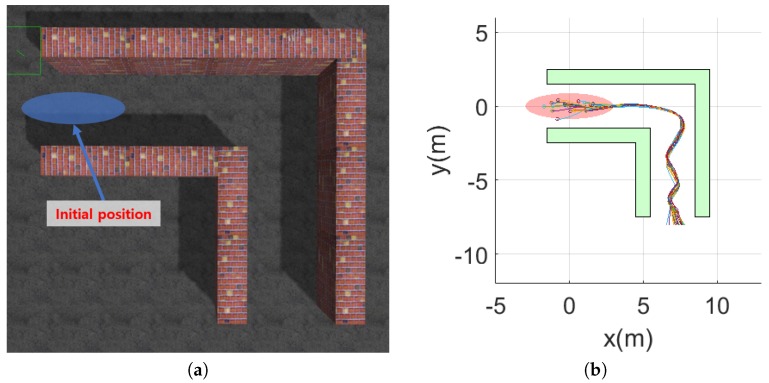

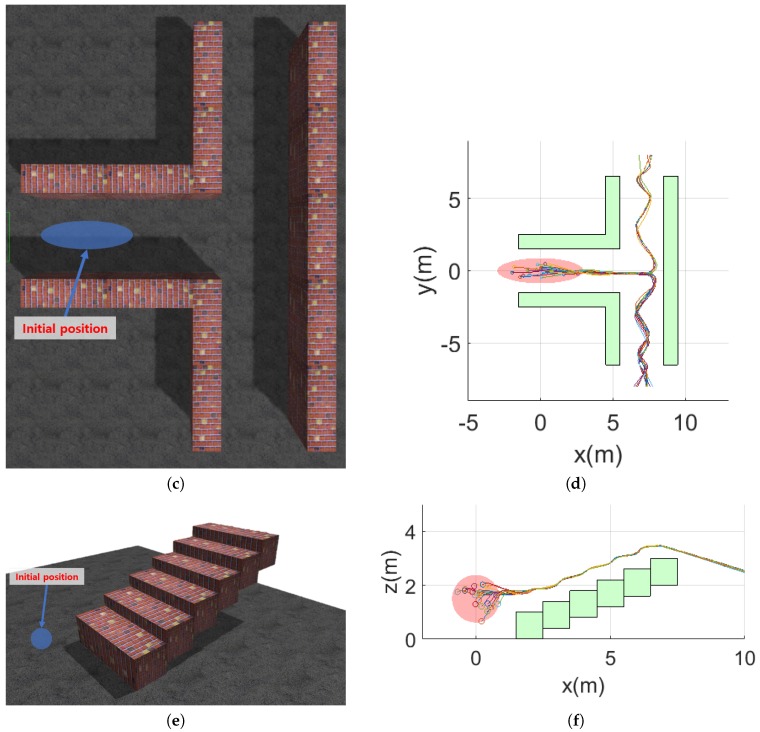
Maps and MAV trajectories for simulations: (**a**) L-shape corner map; (**b**) MAV trajectories at the L-shape corner map; (**c**) T-shape junction map; (**d**) MAV trajectories at the T-shape corner map; (**e**) ramp-shape map; and (**f**) MAV trajectories at the ramp-shape map.

**Figure 9:**
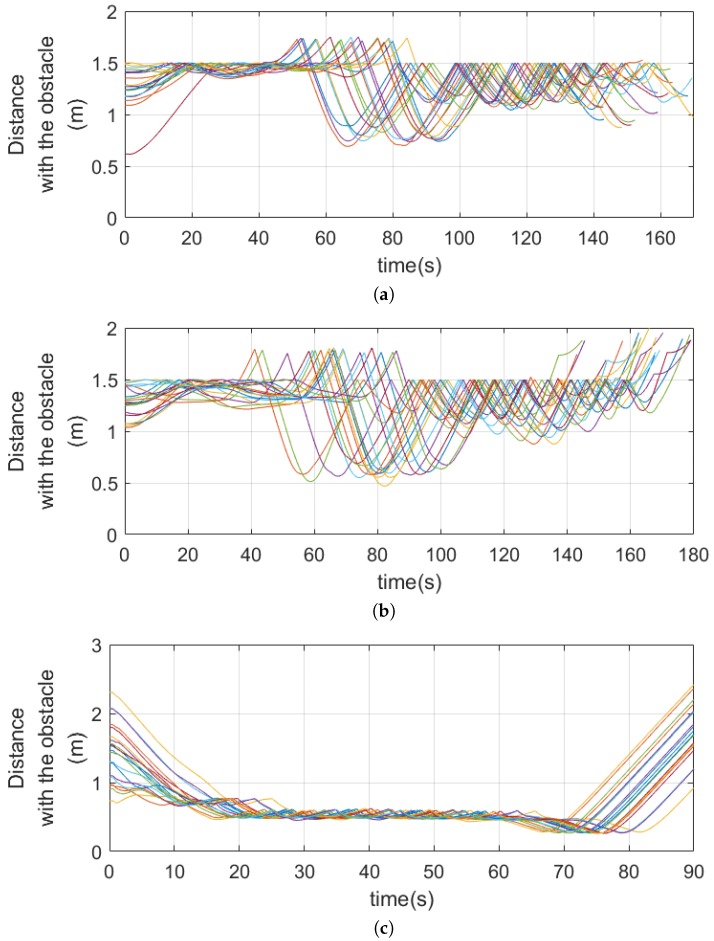
Distance to obstacles in: (**a**) the L-shape corner; (**b**) the T-shape junction; and (**c**) the ramp shape.

Numerical simulations were carried out to verify the performance of the proposed obstacle avoidance strategy. Figure 8 shows the simulation results in L-shape corner, T-shape junction, and ramp-shape maps to verify the horizontal balance, the frontal obstacle avoidance, and the vertical balance strategy, respectively. The first column and second column in Figure 8 show the environment map and the trajectory of the MAV, respectively. For each case, 20 simulations were carried out and the performance of the proposed obstacle avoidance algorithms was analyzed by using the distance to obstacles, as shown in Figure 9. The initial positions were set randomly using the Gaussian distribution with mean and variance set to $x∼N(0,1^{2})$ and $y∼N(0,0.3^{2})$ in the L-shape corner and T-shape junction map and $x∼N(0,0.3^{2})$ and $z∼N(1.5,0.3^{2})$ in the ramp-shape map. Table 1 shows the success rate, minimum distance to the obstacle and standard deviation of minimum distance averaged for 20 simulations. The success rate was defined by considering the size of the MAV. The size of the AscTec Firefly MAV used in this simulation was 0.605 m, 0.665 m, and 0.165 m for *x*, *y*, and *z* direction, respectively. In the case of the L-shape and T-shape maps, the collision of the MAV was determined if an obstacle was within a certain distance from the center of the MAV; the collision boundary was set to 1.5 times of the half of the *y* direction size of the MAV. In the case of the ramp-shape map, the MAV was considered to have a collision if the minimum distance was shorter than 1.5 times of the half of *z* direction size of the MAV.

**Figure 10:**
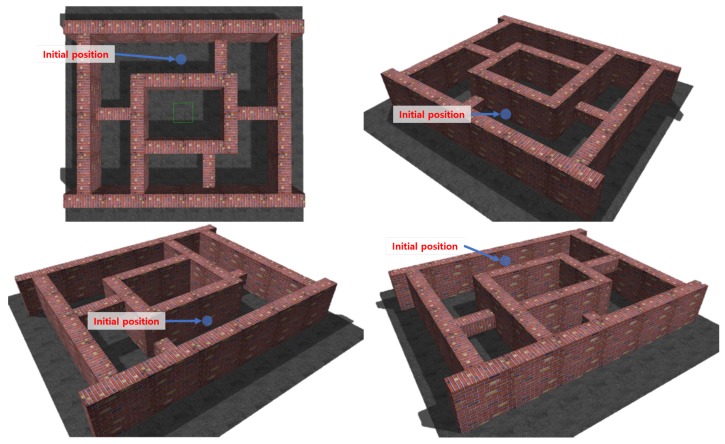
Simulation environment for the integrated obstacle avoidance strategy.

**Figure 11:**
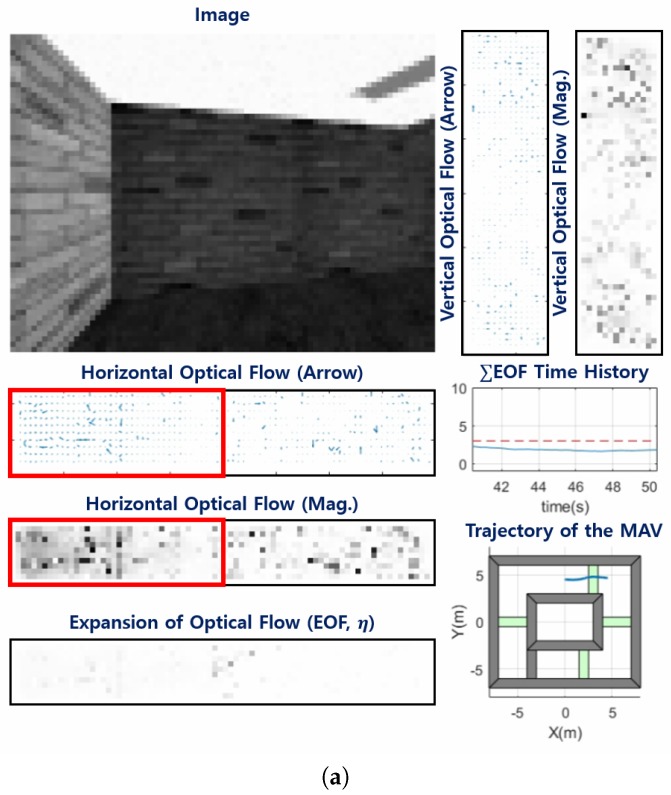

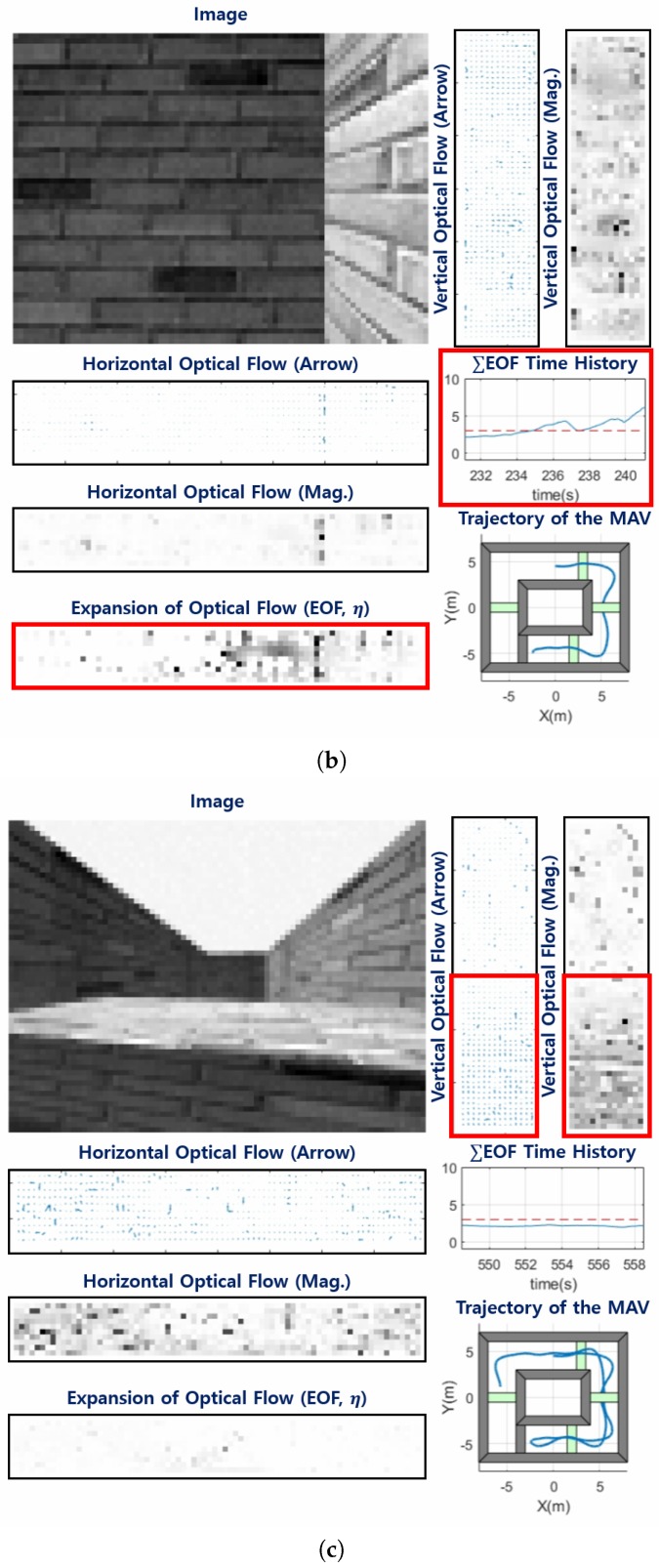

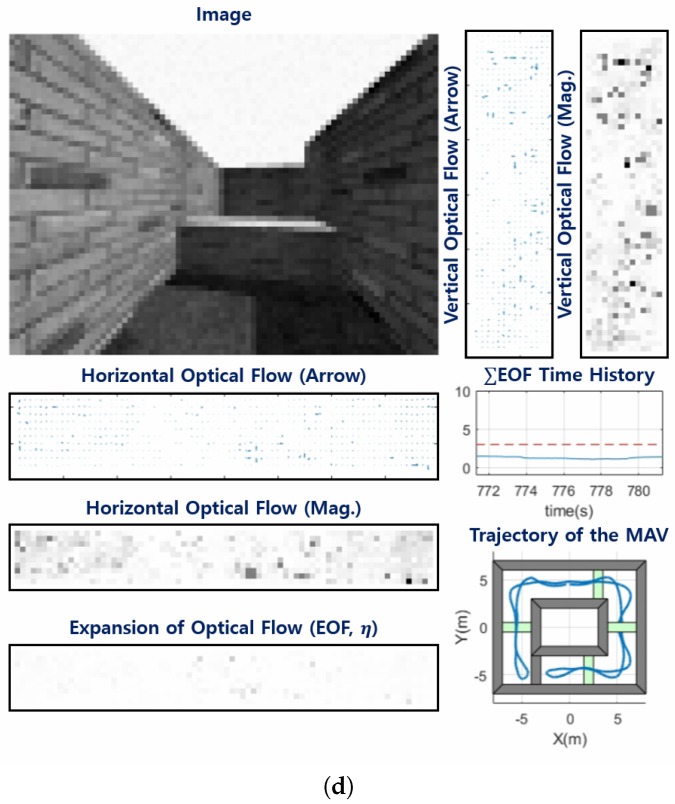
Simulation results for the hybrid obstacle avoidance strategy: (**a**) t = 50 s; (**b**) t = 241 s; (**c**) t = 558 s; and (**d**) t = 781 s.

The hybrid obstacle avoidance strategy was also verified in the complex environment surrounded by walls and containing horizontal columns, as described in Figure 10. Figure 11 shows simulation results such as the image from the camera, optical flows and its magnitude at each window, magnitude of the EOF and its time history, and trajectory of the MAV during the simulation. In the EOF time history, the red dashed line represents where the sigmoid weighting for the obstacle avoidance strategy changed rapidly (i.e., $\eta \geq \eta_{eta,0}=3$). At 50 s, as shown in Figure 11a, the EOF was around 2 and the MAV avoided surrounding walls by using the horizontal balance strategy. At 241 s, as shown in Figure 11b, as the MAV approached the frontal wall, the EOF became large and the frontal obstacle avoidance strategy was dominant, which made the MAV turn sharply. At 558 s, as shown in Figure 11c, as the optical flow in the lower half plane in the vertical optical flow window was bigger than in the upper half plane, the MAV avoided the horizontal column by using the vertical balance strategy. The movie clip for the simulation can be found at https://drive.google.com/file/d/1i9Cx2NoRTqqSM99g8u1YIcXqaS8sRJ8k/view.

### 4.3. Yaw Rate Effect Compensation

**Figure 12:**
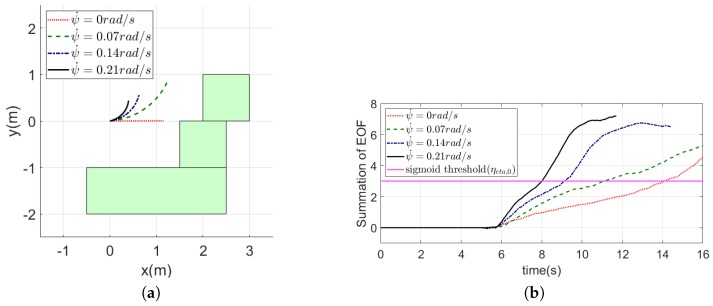
Trajectories of the MAVand the time history of the sum of the EOF when the MAV moves with a constant speed: (**a**) trajectories of the MAV; and (**b**) time history of the sum of the EOF.

The obstacle avoidance strategy used in this research assumes that the MAV moves forward without the significant yaw motion. However, if the MAV meets an obstacle, it will turn to avoid the obstacle. If the yaw rate from turning is too large, then the assumption is no longer valid. In other words, a large yaw rate could generate the unintended optical flow and EOF. Especially, when the MAV encounters a frontal obstacle, if it rotates at a large yaw rate, it would make the EOF larger than expected. To verify the effect of yaw rates on the EOF, an illustrative simulation was conducted. Figure 12a shows the trajectories of the MAV moving forward while the turning with the yaw rate of 0,0.07,0.14, and 0.21 rad/s. The time history of the EOF for each case is shown in Figure 12b. It shows that the larger the yaw rate is, the larger EOF it generates.

To compensate the effect of the yaw rate on the EOF, the yaw rate compensator was added to the frontal obstacle avoidance strategy (Equation (19)) as:

(32) $$\begin{array}{c} {\dot{\psi }}_{d,comp}^{\eta }=sign\left(e_{\eta }\right)\left(k_{P,\eta }\eta_{sum}+k_{D,\eta }{\dot{\eta }}_{sum}+k_{\psi }\dot{\psi }\right), \end{array}$$

where $k_{\psi }$ is a negative gain and $\dot{\psi }$ is a yaw rate of the MAV.

**Figure 13:**
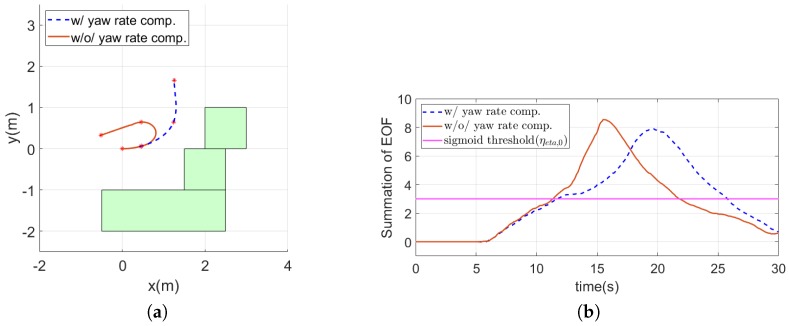

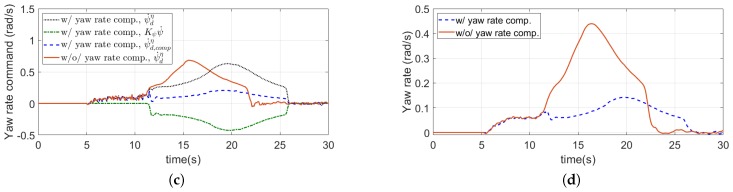
Simulation result of yaw rate effect compensation: (**a**) trajectories of the MAV; (**b**) time history of the sum of the EOF; (**c**) time history of the yaw rate command; and (**d**) time history of the yaw rate.

Figure 13a shows the trajectories of the MAV with and without yaw rate compensation. The star symbols on the trajectories are marked every ten seconds. The MAV with the compensator avoided the obstacles smoothly, whereas the MAV without the compensator took an unexpected U-turn. As shown in Figure 13b, since the summation of the EOF exceeded the sigmoid threshold at about 11 s, the obstacle avoidance strategy was changed from the balance strategy to the frontal obstacle avoidance and the yaw rate compensation was applied. In Figure 13c, the red line indicates the yaw rate command of the MAV without yaw rate compensation (${\dot{\psi }}_{d}^{\eta }$). For the MAV with yaw rate compensation, the green and blue lines represent yaw rate compensation command ($k_{\psi }\dot{\psi }$) and the resultant yaw rate command (${\dot{\psi }}_{d,comp}^{\eta }$), respectively. As the MAV without the compensator approached the obstacles, the EOF and yaw rate increased each other. It resulted in unexpectedly large EOF and yaw rates, as shown in Figure 13d. That is why the MAV without the compensator took the U-turn shown in Figure 13a. On the other hand, the yaw rate compensator alleviated the unexpectedly large yaw rates and EOF as $k_{\psi }\dot{\psi }$ took the opposite sign of ${\dot{\psi }}_{d}^{\eta }$. More rigorous analysis of the effect of the yaw rate on the optical flow remains as future work.

## 5. Indoor Flight Experiments

### 5.1. Experiment Setup

**Figure 14:**
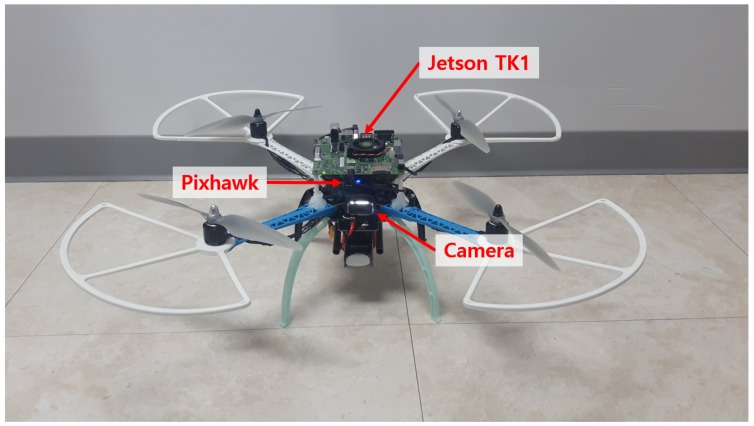
Customized MAV.

**Figure 15:**
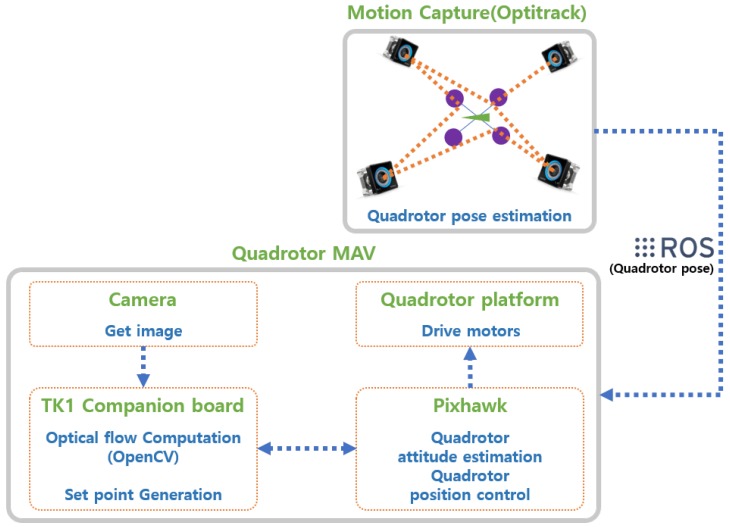
Quadrotor MAV setup and indoor localization system for obstacle avoidance experiments.

**Figure 16:**
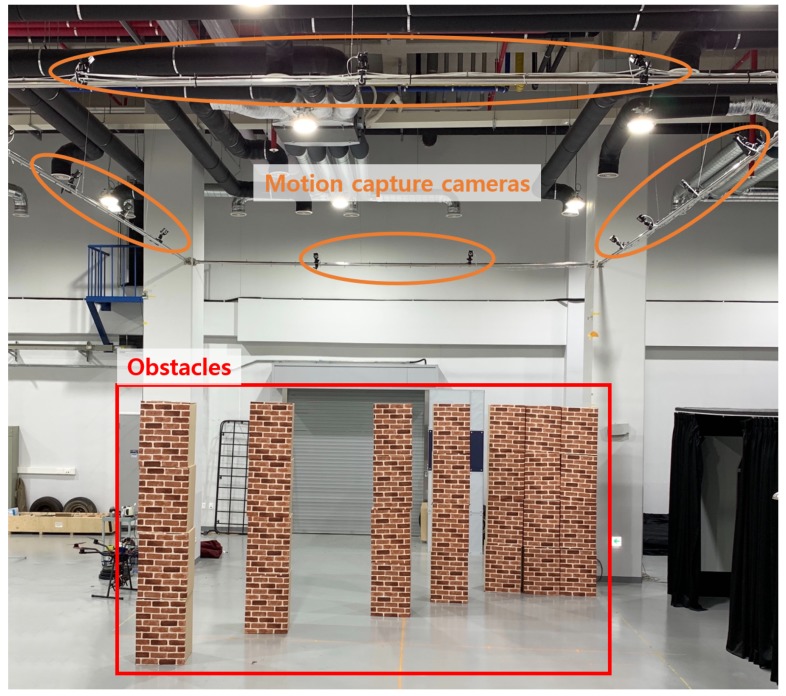
A sample environment for indoor experiments.

The hardware for experiments was configured with the customized MAV, as shown in Figure 14. Figure 15 shows the quadrotor MAV setup and indoor localization system (Optitrack) for obstacle avoidance experiments. The NVIDIA Jetson TK1 board was used as the mission computer for obstacle avoidance. It received the image data from the webcam (LifeCam HD-3000, Microsoft, Redmond, WA, USA) and estimated the optical flow using the sequence of the image data. As described in Section 3.5, the obstacle avoidance algorithm required the current pose data to generate a guidance and control command. To estimate the pose at the indoor environment, the motion capture system was exploited. The position data received by the motion capture system were transmitted to the Pixhawk autopilot and fused with sensors such as barometer and magnetometer. The fused data were used to obtain the six-degrees of freedom states of the MAV. By using the computed optical flow and the MAV states, the MAV generated the guidance input expressed by the desired position and heading angle in the Jetson TK1 board and transmitted them to the Pixhawk autopilot. Then, the Pixhawk autopilot drove the motors so that the MAV moved to the desired position and heading angle. The optical flow was calculated with a sequence of the images at 8 Hz and the obstacle avoidance algorithm was run at 1 Hz. Figure 16 shows a sample environment for indoor environments where obstacles were set by brick-patterned boxes so that the optical flow could be generated easily from obstacles.

### 5.2. Experiment Results

**Figure 17:**
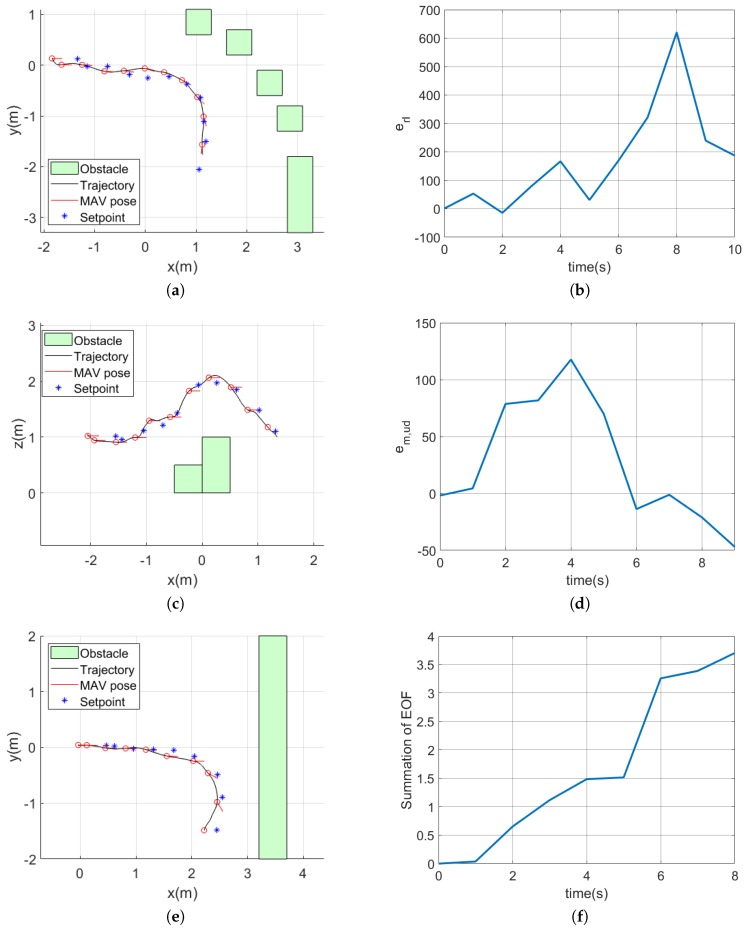
Trajectories of the MAVand time history of the optical flow difference or the sum of the EOF: (**a**) trajectory for the horizontal balance strategy experiment; (**b**) time history of the optical flow difference for the horizontal optical flow experiment; (**c**) trajectory for the vertical balance strategy experiment; (**d**) time history of the modified optical flow difference for the vertical balance strategy experiment; (**e**) trajectory for the frontal obstacle avoidance strategy experiment; and (**f**) time history of the summation of the EOF for the frontal obstacle avoidance strategy experiment.

To verify obstacle avoidance strategies in a real world, indoor flight experiments were conducted. The waypoint guidance strategy was applied for maintaining the reference height when there was no vertical obstacle around. Figure 17 shows the position of obstacles (green boxes), trajectory of the MAV (black line) and desired position (blue star marks). In the trajectory, the red circles are marked every 1 s. The red circle also means the time that a new control input was generated. In the first environment, the optical flow difference between the left and right half planes was always positive and thus the MAV turned to the right as the MAV went forward. In the second environment, the obstacle was placed in a two-step stair shape. As the optical flow was large at the lower half plane around 1–6 s, as shown in Figure 17d, the MAV increased the height continuously to avoid the obstacle vertically. After passing the obstacle, there was a small difference of the optical flow between upper and lower half planes and the MAV returned to the reference height. In the last environment, the wall-like obstacle existed in front of the MAV. After 5 s, the sum of the EOF increased as the MAV moved towards the wall; as a result, the MAV turned the heading angle abruptly using mainly the frontal obstacle avoidance strategy.

## 6. Conclusions and Future Work

This paper proposes the vision-based obstacle avoidance strategy using the optical flow which can be used in various 3-D textured environments. In particular, it exploits the EOF to cope with the front obstacle efficiently along with avoiding obstacles horizontally and vertically. To verify the performance of the proposed approach, numerical simulations and indoor flight experiments were carried out. The proposed obstacle avoidance strategy using optical flows requires a light computational power; hence, it could be readily applicable to miniaturized MAVs requiring the lightweight CPU such as insect-inspired flapping aerial vehicles. It is worth noting that the use of obstacle avoidance based on optical flows is limited to only relatively well-textured environments since optical flows are rarely generated on textureless objects such as white walls, wires and poles.

As the forward speed of the MAV is assumed to be kept relatively slow in our approach, the effect of pitch control on the optical flow computation was negligible. However, for high speed maneuvers, the effect of pitch control on the optical flow would be significant; this will be dealt with in the future work. Besides, although this study proposed the yaw rate effect compensator in the situation where the MAV encounters the frontal obstacle, more rigorous analysis and experiments need to be performed. For general environments, other vision-based algorithms using feature or color detection could be combined with the proposed optical flow-based approach, which remains as future work.

## Figures and Tables

**Table 1:** Simulation results of obstacle avoidance for three types of map (SD, standard deviation of minimum distance with 20 simulations).

Map	L-Shape	T-Shape	Ramp-Shape
Success rate [%]	100	95	100
Minimum distance [*m*]	0.62	0.47	0.34
SD	0.0747	0.0583	0.0108

## Abbreviations

The following abbreviations are used in this manuscript:

MAV	Micro Aerial Vehicle
PD	Proportional-Derivative
ROS	Robot Operating System
FOE	Focus Of Expansion
EOF	Expansion of the Optical Flow
VI	Visual-Inertial
SD	Standard Deviation
